# Angiosarcome de l´épaule révélé par un ancien traumatisme: à propos d´un cas et revue de la littérature

**DOI:** 10.11604/pamj.2020.36.40.20751

**Published:** 2020-05-27

**Authors:** Manix Ilunga Banza, Israël Badypwyla Tshiamala, Augustin Kibonge Mukakala, Christelle Ngoie Ngoie, Néron Tapenge Shutsha, Stéphane Ilunga Mukangala, Vincent De Paul Kaoma Cabala, Mireille Meuke Tchankui, Trésor Kibangula Kasanga, Nathalie Dinganga Kapessa

**Affiliations:** 1 Département de Chirurgie des Cliniques Universitaires de Lubumbashi, Université de Lubumbashi, Province du Haut-Katanga, Ville de Lubumbashi, République Démocratique du Congo; 2 Service de Chirurgie de l´Hôpital Jason Sendwe, Province du Haut-Katanga, Ville de Lubumbashi, République Démocratique du Congo

**Keywords:** Angiosarcome, épaule, traumatisme, Angiosarcoma, shoulder, trauma

## Abstract

L´angiosarcome est une tumeur rare à multiples localisations mais celle cutanée est la plus fréquente faisant de sa symptomatologie un polymorphisme clinique. C´est une tumeur de mauvais pronostic à cause de sa grande propension à la récidive locale et aux métastases à distances précoce. Nous rapportons un cas de découverte fortuite d´un angiosarcome de l´épaule sur un ancien foyer de traumatisme direct datant d´une année chez un patient de 72 ans, venu consulter pour douleur persistante en regard d´une tuméfaction à la face postérieure de l´épaule contemporaine au traumatisme. L´examen clinique conclu en un hématome ancien partiellement calcifié soutenu par la ponction de la masse ayant ramené 5 cc de sang d´aspect noirâtre et renforcé par une échographie. La radiographie de l´épaule n´étant pas pathologique, une exploration de la tuméfaction a été entreprise. Celle-ci a permis de mettre en évidence et reséquer des tissus friables, d´aspect rougeâtre, avec un saignement important difficilement maitrisé pendant deux jours compliqué d´une anémie non tolérée corrigée par deux transfusions sanguines. L´anatomie pathologique des tissus réséqués a conclu en un angiosarcome moyennement différentié avec malheureusement des berges non saines. Un bilan d´extension a été réalisé à la recherche des métastases. Pas de récidive locale sur trois mois et le patient a été transféré dans un centre spécialisé à Lusaka pour la radiothérapie complémentaire. L´objectif du présent travail est de présenter un cas rare d´angiosarcome de découverte fortuite sur un antécédent de traumatisme de l´épaule gauche et de ressortir les aspects thérapeutiques en passant en faisant une revue de la littérature.

## Abstract (en)

Angiosarcoma is a rare tumor which can affect multiple sites. However, it most commonly arises from the skin. Then symptoms have been associated with clinical polymorphism. This tumor has a poor prognosis due to its high tendency to local recurrence and distant metastases. We here report the case of a 72-year old man in whom an angiosarcoma of the shoulder arose from a trauma which occurred one year before it was accidentally detected. The patient presented with persistent pain in a swelling area at the posterior portion of the shoulder involved in the trauma. Clinical examination showed previous and partially calcified hematoma. Diagnosis was based on puncture biopsy of the mass collecting 5 cc of blackish blood and on ultrasound. Shoulder x-ray was normal. An assessment of the swelling was performed. This helped to highlight and resect some reddish, significantly bleeding friable tissue hardly managed for two days and complicated by poorly tolerated anemia corrected with two blood transfusions. Anatomopathological examination of the resected tissues showed moderately differentiated angiosarcoma unfortunately without healthy margins. Staging evaluation was performed in order to detect metastases. No local recurrence was reported over a period of three months and the patient was referred to a specialized center in Lusaka for complementary radiotherapy. The purpose of this work is to report a rare case of angiosarcoma accidentally detected in a patient with a history of trauma to the left shoulder and to highlight the therapeutic features, while conducting a literature review.

## Introduction

L´angiosarcome est une tumeur maligne peu commune, rare, dérivée des cellules endothéliales des vaisseaux sanguins [1, 2]. L´angiosarcome peut se localiser dans n´importe quelle région de l´organisme [2]; sa localisation prioritaire étant la peau et les tissus mous [3], préférentiellement au niveau de la face et du cuir chevelu (scalp) des personnes âgées [1, 2]. Cependant, plusieurs autres localisations sont décrites dans la littérature notamment l´oreille, le foie, la rate, le rein, le cœur [4-6]. Les angiosarcomes sont des tumeurs malignes rares avec une évolution péjorative et un mauvais pronostic [7, 8] car ils développent une récidive locale et des métastases à distance précocement dont il est difficile de prévenir l´évolution [8]. Ils ne représentent que 1 à 2% des sarcomes des tissus mous et moins de 1% des tumeurs malignes [7]. Les facteurs de risque connus sont le lymphœdème chronique, congénital, traumatique, et sont impliqués dans plus de 10% des angiosarcomes des membres, les radiations ionisantes; ils se produisent le plus souvent dans les zones de la peau exposées à long terme au soleil chez les personnes âgées; exceptionnellement les angiosarcomes peuvent se développer sur un angiome pré-existant [9]. C´est une tumeur beaucoup plus observée chez les personnes âgées, les hommes étant deux fois plus touchés que les femmes [10]. L´anatomie pathologique reste le garant du diagnostic de certitude. Les angiosarcomes vont d´une tumeur hautement différentiée ressemblant à l´hémangiome, à une tumeur anaplasique difficile à distinguer d´un carcinome [11]. Les formes moyennement et bien différentiées sont caractérisées par la présence des cavités vasculaires irrégulières, qui dissèquent le collagène et réalisent un réseau anastomotique, bordé des cellules à noyaux augmentés de volume et souvent hyperchromatiques [9]. Les marqueurs endothéliaux incluent CD31, CD34, et le facteur de Von Willebrand, sont souvent positivement exprimés par l´immunohistochimie [8]. C´est une pathologie grave dotée d´un pronostic péjoratif et d´un taux de survie à 5 ans entre 12 et 24% [12]. Les modalités de traitement de l´angiosarcome incluent la chirurgie, la radiothérapie, l´immunothérapie et la chimiothérapie [8]. En cas des lésions primaires, la chirurgie radicale et la radiothérapie post-opératoire sont suggérées; malheureusement l´obtention des berges saines avec marge de sécurité est rarement retrouvée pour l´angiosarcome malgré les larges résections en surface [1, 13].

## Patient et observation

**Figure 1:**
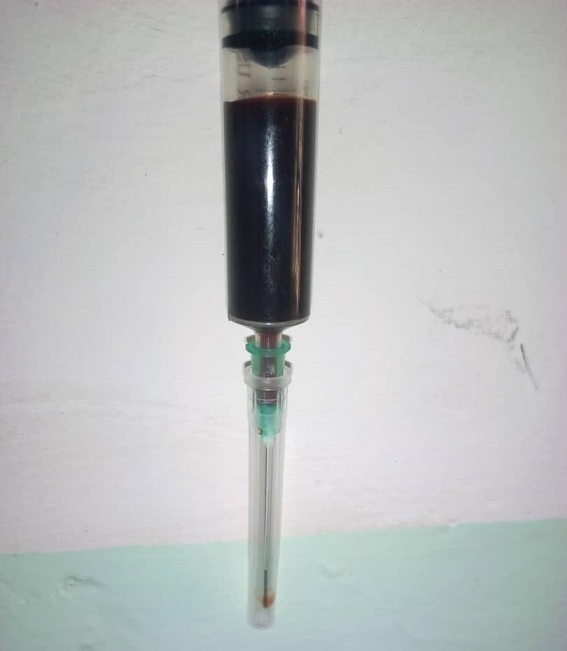
Seringues de 5cc remplie de sang recueillis par ponction en pleine masse

Nous avions examiné en date du 02 juin 2019 un patient âgé de 72 ans, venu consulter pour douleur au niveau de l´épaule gauche. L´histoire révèle qu´il y a une année le patient aurait subi un traumatisme direct sur l´épaule concernée au décours d´une dispute avec un agent de l´ordre qui lui aurait donné un coup de matraque sur l´épaule concernée. Les suites post-traumatique serait marqué d'une tuméfaction et une vive douleur dans la dite région. Il aurait consulté un centre médical ou des antalgiques lui auraient été administré pendant une semaine sans rémission complète des signes. La douleur aurait persisté pendant 11 mois et aurait même entrainé une diminution de la mobilité de l´épaule malgré les multiples consultations et automédications aux antalgiques usuels. En dehors de cet antécédent traumatique, aucun autre antécédent n´était contributif (pas d´hypertension artérielle (HTA), pas de diabète, pas de gastrite, pas d´antécédent toxico-allergique). L´examen général était dans les limites de la normale. L´examen locorégional avait noté une légère tuméfaction dans la région postérieure de l´épaule gauche. Cette tuméfaction était ovoïde, d´axes mesurant 5x6 cm, pas de rougeur visible en regard. A la palpation, son aspect était ferme avec des foyers de rénitence par endroit, sensible, se laissant déprimer, pas de chaleur à la palpation de la masse, pas de souffle perçu à l´auscultation. La mobilité de l´épaule était conservée. La masse ne semble pas en rapport avec l´omoplate. La pauvreté des signes cliniques a fait suspecter le diagnostic d´un ancien hématome partiellement calcifié. C´est ainsi qu’une ponction exploratrice a été faite à l´aide d´une aiguille de 5cc au pôle anti-déclive; cette dernière a ramené du sang non coagulable, d´aspect noirâtre (Figure 1) faisant ainsi poser le diagnostic clinique d´un ancien hématome.

**Figure 2:**
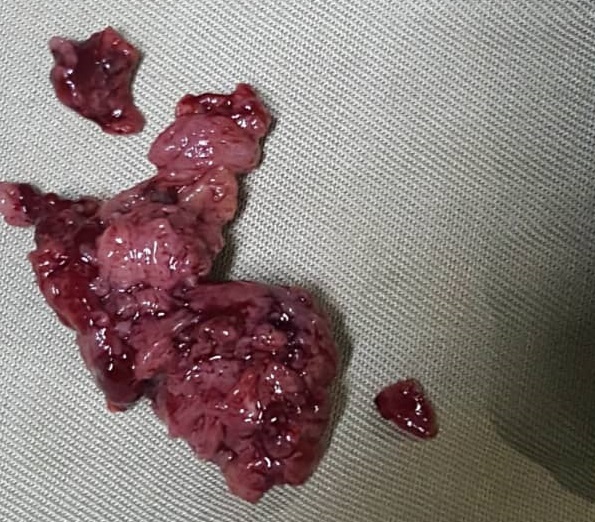
Masse extirpée faite des tissus fiables, rougeâtre, et très hémorragique

**Figure 3:**
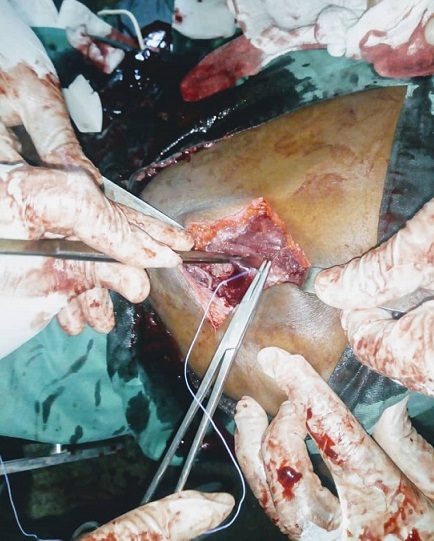
Plaie opératoire entrain d´être refermée après extirpation de la tumeur: les berges de la plaie semblent macroscopiquement saines

Une radiographie de l´épaule et une échographie avaient ainsi été réalisées. La radiographie a exclu une atteinte osseuse et a montré une image légèrement opaque dans les parties molles tandis que l´échographie a rapporté des lésions hétérogènes, d´écho-structure tissulaire avec des plages liquidiennes témoignant la présence des phénomènes nécrotiques ou hémorragiques évoquant un diagnostic probable d´hématome organisé. Au vu de tous ses éléments clinique et paraclinique, une évacuation de l´hématome a été décidée. Un bilan hématologique pré-opératoire a été réalisé comprenant: hémoglobine: 13 g/dl; hématocrite: 41%; groupe sanguin: A; rhésus: positif; temps de saignement: 2 minutes 30 secondes; temps de coagulation: 4 minutes; HIV: négatif. Au cours de l´exploration, la mise en évidence dans les parties molles tant sus et sous aponévrotiques d´un tissu rougeâtre, mollasse et friable, et surtout très hémorragique a été observé et extirpé presqu´en totalité (Figure 2). L´hémostase a été extrêmement difficile a réalisé malgré la présence du bistouri électrique. La plaie a ainsi été suturée après avoir extirpé toute la masse (Figure 3) qui a ainsi été apporté au service d´anatomopathologique pour le diagnostic histologique. Les suites post-opératoires ont été compliquées d´une importante hémorragie ayant entrainée d´abord une ré-exploration de la plaie pour assurer l´hémostase; et deux transfusions ont été administré suite à une anémie décompensée avec un changement de pansement plusieurs fois par jour pendant les 72 heures post-opératoires avec des pansements compressifs. C´est au bout de 3 jours que l´hémostase a ainsi été maitrisée complètement avec des pansements de la plaie opératoire devenus secs et propres. Les résultats de l´analyse histologique ont donné comme résultat: présence de structures vasculaires de taille et de forme irrégulières, disséquant le collagène, le tissu adipeux, et communiquant entre elles, réalisant un réseau anastomotique. Ces structures vasculaires sont bordées des cellules, à noyaux augmentés de volume, souvent hyperchromatiques, réalisant souvent des structures papillaires endoluminales. Conclusion: images suggestives d´un angiosarcome moyennement différencié (grade 2). L´immunohistochimie s´avère utile.

Malheureusement, la non disponibilité de l´immunohistochimie sur toute la ville explique sa non réalisation. Après réception des résultats d´anatomie pathologique, un bilan d´extension a ainsi été réalisé à la recherche des probables métastases. Le bilan d´extension réalisé incluait un examen physique minutieux à la recherche des métastases cutanées et une imagerie comprenant une radiographie thoracique, une radiographie du crâne et de la colonne vertébrale, une échographie abdominale et pelvienne sans aucune anomalie retrouvée. C´est ainsi qu´après un suivi de 3 mois sans aucune récidive locale, un transfert dans un centre spécialisé de radiothérapie à Lusaka a été fait pour améliorer l´efficacité du traitement de l´angiosarcome chez notre patient.

## Discussion

Les angiosarcomes cutanés sont rares, et représentent 4-5% des sarcomes cutanés et moins de 1% de tous les sarcomes [5]. Pendant 35 ans soit de 1955 à 1990, 67 patients porteurs d´angiosarcome de la tête et du cou seulement ont été colligés par Mark à l´université de Californie [1], justifiant sa rareté. Et dans notre milieu, c´est une qui est encore d´une extrême rareté. C´est une tumeur qui touche beaucoup plus les adultes. Dans la série de Aust MR portant sur 32 patients, l´âge moyen était de 63 ans [2] alors qu´il était de 71 ans dans celle de Pawlik [13] et de 60 ans dans celle de Penel [14]. Notre patient était âgé de 72 ans ceci corrobore ainsi avec les diverses données de la littérature ou la majorité des cas rapportés sont des adultes. Le sexe masculin de notre patient ne peut confirmer la prédominance du sexe masculin quoique les rares séries des cas publiées confirment une prédominance masculine 23 hommes pour 9 femmes dans la série de Aust [2] et 69% d´hommes dans celle de Andrea Claudia [15]. Par rapport aux antécédents, notre patient avait un antécédent de traumatisme de l´épaule concernée une année avant. Cependant, nous ne pouvons pas affirmer que cet antécédent serait la cause de l´angiosarcome quoique le traumatisme est également cité parmi les facteurs de risque de survenue des angiosarcomes [9] et Mazahiro [8] affirme que son patient de 73 ans avec angiosarcome de la tête (scalp) comme une lésion primaire avait un antécédent de traumatisme de scalp ayant causé un angiome. La présentation clinique est polymorphe en fonction de la localisation de la tumeur. Celle viscérale peut se manifester par une hémorragie digestive [16], le plus souvent la tumeur cutanée se manifeste par une tuméfaction comme dans notre cas ou la tuméfaction était localisée à la face postérieure de l´épaule gauche, rénitente par endroits, légèrement sensible alors que l´articulation de l´épaule était libre des mouvements. La tuméfaction a été aussi la manifestation clinique principale chez Karima Issara [5] au niveau de l´oreille sous forme de masse bourgeonnante, ferme, indolore, sans signes inflammatoires. Cependant dans notre cas, la tuméfaction était ovoïde de 5x7 cm, douloureuse mais n´était pas bourgeonnante sans aucun caractère inflammatoire.

Il s´agit d´une tumeur dont le pronostic est généralement mauvais, doté d´un fort potentiel des métastases [1], surtout pulmonaires [4, 14]. La fréquence des métastases pourrait atteindre 50% des cas d´angiosarcomes [5], le faisant ainsi considéré comme une maladie systémique avec un taux de survie souvent faible surtout en cas de forme métastasée ou la survie moyenne varie en fonction des auteurs allant de 7 mois sans traitement à 20 mois après chirurgie et radiothérapie [9], de moins de 11 mois [14], de 28,4 mois [13], 24% à 2 ans [1] et à 5 ans la survie est de l´ordre de 12 à 24% [12] allant jusqu´à 44% [1]. Dans la série de Mark [1], 52 cas sur les 67 colligés ont récidivés après le traitement primaire, 28 patients ont eu des métastases à distance; ce qui confirme le caractère péjoratif du pronostic de cette pathologie. Chez notre patient, malgré le manque de récidive sur un recul de 3 mois, nous ne saurons affirmer cette théorie du fait d´un suivi cours de seulement 3 mois avant le transfert de notre patient en Zambie pour la radiothérapie. Le suivi doit être assez long pour confirmer ces théories, il a été en moyenne de 2,1 ans chez Aust avec des extrêmes de 83 jours à 9,7 ans [2], de 30 mois chez Mark RJ pour des extrêmes de suivi de 1 à 173 mois [1]. La régression spontanée d´un angiosarcome est un phénomène rare [8]. Les dimensions de la tumeur sont donc des données très importantes et conditionnerait non seulement le pronostic mais aussi les modalités de prise en charge. La tumeur de moins de 7,0 cm de diamètre et envahissant uniquement le tissus cellulaire sous cutané ont un meilleur taux de survie par rapport aux autres. Les patients avec masse de moins de 1,5 cm ont été traités avec succès par la chirurgie seule. Ceux traités par la chirurgie puis la radiothérapie ont un meilleur pronostic également [2].

Mark RJ souligne que le traitement optimal n´est pas bien défini [1], cependant, beaucoup d´auteurs s´accordent sur la pertinence de la chirurgie qui est quasi obligatoire en cas d´angiosarcome. Cependant, l´éxérèse chirurgicale doit être la plus large possible avec des marges de 2-5 cm en restant compatible avec une qualité de vie acceptable [10]. Malheureusement, les larges marges chirurgicales sont souvent difficile à obtenir vu la localisation de ces tumeurs, dans la plupart des séries des cas, seules les petites tumeurs (moins de 5 cm), de bas grade et avec une résection complète microscopique peuvent bénéficier d´une chirurgie seule [15]. Dans la série de PAWLIK [13], 96,6% des patients ont subi la chirurgie; ceci justifie également notre traitement chirurgical. La chirurgie reste donc la première option de traitement des patients avec angiosarcome, l´obtention des berges saines (marge de sécurité) souvent difficile à obtenir, surtout au niveau du scalp où dans sa série seulement 21,4% des prélèvement avaient des marges saines [1, 13]. La radiothérapie était associée significativement à une baisse du taux de décès (p = 0,006) et doit être employée de façon routinière et augmenterait le taux de survie [13]. Cependant, la survie médiane à 32 mois serait meilleure pour la combinaison chirurgie-radiothérapie par rapport à la chirurgie seule [1, 17]. Cette observation est également rapporté par LEIGHTON que l´association de la radiothérapie à la chirurgie offre de meilleurs chances de contrôle local par rapport à une chirurgie seule, même après une résection complète [18]. Ceci a justifié notre prise en charge de préconiser une radiothérapie en complément de la chirurgie en transférant le patient à Lusaka dans une unité de radiothérapie. La chimiothérapie est le traitement de choix d´un angiosarcome métastatique [4, 19] et réservée aux formes non opérables [4]. Les molécules utilisées sont la Doxorubicine (traitement de base) qui constitue la première ligne de traitement antimitotique, Paclitaxel (taxol) [14]. Quelques cas de régression spontanée de la tumeur primitive et des métastases sous chimiothérapie sont décrit dans la littérature [8].

## Conclusion

Les angiosarcomes cutanés sont rares mais sont cependant de mauvais pronostic à cause du risque élevé de récidive locale mais surtout de métastases à distance notamment pulmonaires. La chirurgie reste la base du traitement mais son association avec la radiothérapie améliore la survie du patient.

## Conflits d’intérêts

Les auteurs ne déclarent aucun conflit d´intérêts.
